# Sleep Deprivation, Sleep Disorders, and Chronic Disease

**DOI:** 10.5888/pcd20.230197

**Published:** 2023-08-31

**Authors:** Alberto R. Ramos, Anne G. Wheaton, Dayna A. Johnson

**Affiliations:** 1 Department of Neurology, University of Miami, Miller School of Medicine, Miami, Florida; 2 Division of Population Health, National Center for Chronic Disease Prevention and Health Promotion, Centers for Disease Control and Prevention, Atlanta, Georgia; 3 Department of Epidemiology, Rollins School of Public Health, Emory University, Atlanta, Georgia

This editorial aims to highlight the complex interplay among sleep, mental health, and chronic disease, emphasizing the critical role that sleep plays in health outcomes and overall well-being. With the mounting evidence linking sleep to numerous health problems — from mental health disorders to chronic diseases — it is paramount that we shift our focus toward understanding sleep not as a passive state but as a vital process for brain restoration and regulation. Recognizing and addressing sleep disturbances and disorders, along with promoting comprehensive strategies for improving sleep health, is a national imperative with far-reaching economic and health implications. 

The articles in this collection in *Preventing Chronic Disease *(PCD) — Sleep Deprivation, Sleep Disorders, and Chronic Disease — provide valuable insights into the bidirectional relationships between sleep, mental health, and chronic disease throughout the lifespan. Furthermore, the articles shed light on key themes — starting from childhood to young adulthood — while considering the role of parents and sociodemographic factors, the effect of sleep health on various racial and ethnic groups, and the geographic variation in the prevalence of short sleep duration.

The relationships among sleep, mental health, and chronic disease have garnered considerable attention in recent years (1). Studies consistently highlight the association between 1) dimensions of sleep and sleep disorders and 2) mental, behavioral, and developmental disorders. Stemming from this research, public health awareness of the importance of sleep has increased, as highlighted in *Healthy People 2030* goals (2). In June 2022, the American Heart Association added sleep duration as a vital component of its Life’s Essential 8 (an update from Life’s Simple 7) as a metric for cardiovascular health (3). In addition, the American Academy of Sleep Medicine (AASM) and the Sleep Research Society jointly released a consensus statement in 2015 recommending the amount of sleep for healthy adults (4). This statement was followed by the AASM’s consensus statement in 2016, which provides sleep duration recommendations for pediatric populations (5). Similarly, an expert panel convened by the National Sleep Foundation made evidence-based recommendations on sleep duration for various age groups, from 14 to 17 hours for newborns to 7 to 8 hours for older adults (6). The American Academy of Pediatrics supports the delay of school start times for adolescents to ensure they receive adequate sleep (7). The Canadian 24-Hour Movement Guidelines for Children and Youth acknowledged the importance of healthy sleep, integrating sleep recommendations along with guidelines for physical activity and sedentary behavior (8). These developments highlight the growing recognition of sleep’s crucial role in overall health and well-being.

Despite this focus on healthy sleep, a substantial proportion of adults in the US fails to meet the recommended hours of sleep; thus, improving sleep is a national imperative with substantial economic and health implications (9). Growing research indicates that racial and ethnic minority groups are disproportionately affected by sleep and circadian disparities, which exacerbate chronic disease disparities (10,11). Today’s 24-hour lifestyle, coupled with the pervasive use of electronics and social media, has normalized inadequate sleep among many children and adolescents, with uncertain effects on brain development, mental health, and vascular health (12). Additionally, emerging evidence links sleep deprivation to adverse cardiometabolic health and cognitive health and an increased risk of dementia among older adults — making it an important acquired risk factor in the 21st century (13). Thus, sleep may be key to reducing the burden of chronic diseases.

Sleep is far from a passive state; it is a vital process for brain restoration and regulation. Inadequate sleep disrupts critical neural processes and impairs cognitive functioning (14,15). Altering these processes provides a mechanistic link through which insufficient sleep contributes to the onset or worsening of mental health, brain disorders, and chronic diseases. Only by bridging the gap between sleep and these important health outcomes can we develop integrated, comprehensive strategies to address the problems related to insufficient sleep. In addition, a growing body of research suggests that insufficient sleep plays a substantial role in the development and worsening of many chronic diseases (3,12,13).

Several studies in this PCD collection demonstrate that insufficient sleep is prevalent among children and adolescents and is associated with mental, behavioral, and developmental disorders. Claussen et al (16) reported that short sleep duration, defined as less than the recommended amount of sleep for one’s age, was more prevalent among children with these disorders, children from racial and ethnic minority groups, and children from households with low socioeconomic status. Prevalence of short sleep duration was associated with inconsistent bedtimes, poor parental mental and physical health, and adverse childhood experiences. Addressing these factors may improve children’s sleep and promote healthy development, particularly among children with low socioeconomic status or from racial and ethnic minority groups (16).

Bird et al (17) demonstrate that parents and caregivers play a crucial role in promoting healthy sleep behaviors in children. We need collaborative efforts between schools and parents to improve child sleep health. Evidence supports engaging parents in the school community and addressing their concerns about sleep promotion initiatives to foster a supportive environment (17).

Insufficient sleep among adolescents is associated with poor mental health, including depressive symptoms and suicidal thoughts. The study in this collection by Gunderson et al (18) analyzed data from the 2021 Florida Youth Risk Behavior Survey and showed that high school students reporting insufficient sleep (<8 hours of sleep on an average school night) were more likely to experience feelings of sadness or hopelessness, consider suicide, and make suicide plans compared with those with sufficient sleep (after adjustment for sex, race and ethnicity, and grade level) (18). These results underscore the importance of addressing sleep as a modifiable risk factor in adolescent mental health and incorporating it into suicide prevention efforts (18).

The COVID-19 pandemic has brought attention to the effect of sleep on mental health. Sliwa et al (19) used data from the Adolescent Behaviors and Experiences Survey, a one-time national survey of high school students during the pandemic, and found that a significant proportion experienced short sleep duration, which was associated with both poor mental health and increased difficulty in doing schoolwork during the pandemic compared with before the pandemic (19). Students who reported less than 7 hours of sleep or poor mental health had a higher prevalence of increased trouble with schoolwork. If we create a comprehensive strategy that incorporates sleep duration, we can better support student mental health and academic achievement.

Researchers also observed an association between sleep and mental health among older students. In a survey of college students by Mbous et al (20), one-quarter of the study population experienced insomnia, which was significantly associated with mental health conditions, specifically attention deficit hyperactivity disorder (ADHD) and depression (20). The odds of insomnia were higher among students who had depression, had symptoms of ADHD, and were employed. Tailored sleep education interventions, focusing on employed students and people with mental illnesses, can help target insomnia symptoms and severity among college students.

Sleep health is a multifaceted concept of sleep–wakefulness patterns tailored to personal, societal, and environmental needs, and it promotes overall well-being. Sleep health is not solely about getting the right amount of sleep but also encompasses the timing, regularity, satisfaction, and efficiency of sleep (21). Sleep is influenced by a mix of biological and environmental factors, often in line with major life events, health issues, lifestyle choices, and sociodemographic factors (21).

Although these various dimensions of sleep are associated with many poor health outcomes, how they are connected is not always clear. Inadequate or disturbed sleep may sometimes contribute to other health conditions and vice versa. Morey et al (22) explored how sleep disturbance could play a mediating role between stress and self-rated health among Chinese and Korean immigrants. Their mediation analyses concluded that 15% to 22% of the associations between stress (perceived stress and acculturative stress, respectively) and self-rated health was attributable to sleep disturbance — suggesting sleep is a key factor in the stress–health relationship (22).

In 2020, one-third of US adults reported short sleep duration, with differences across sociodemographic characteristics and geographic areas. Pankowska et al (23) found that urban–rural differences exist — the prevalence of short sleep duration was lowest among adults living in urban (metropolitan) counties and was higher in micropolitan and rural counties (23). In addition, county-level data showed that counties in the Southeast and along the Appalachian Mountains had a higher prevalence of short sleep duration. These geographic patterns of short sleep duration partially reflect patterns of other chronic conditions. Overall, these findings suggest that incorporating neighborhood-level data and context is crucial for effective local interventions to help US adults get adequate sleep (23).

The articles in this PCD collection offer a wealth of practical information aimed at improving sleep health for the individual and in the community while also recognizing the multifactorial influences, bidirectional relationships, and individual variations in sleep health. By viewing the findings from diverse studies, we can foster a nuanced understanding and develop comprehensive approaches to address the intertwined aspects of sleep, mental health, and chronic diseases. Effectively addressing mental health requires a comprehensive approach, encompassing both sleep disturbances and underlying psychosocial factors. Furthermore, contextual elements such as cultural norms, work demands, and lifestyle constraints substantially affect sleep duration and quality. Future studies should consider tailored approaches and personalized interventions to address these individual variations and contextual factors, thereby optimizing sleep and mental health.

Most of the articles in this collection focus on sleep duration and sleep disturbances, highlighting the need for further research on other aspects of sleep, particularly sleep timing or schedules. Efforts are underway to expand our understanding of sleep beyond sleep duration and disturbances to gain insights into how sleep affects overall health. The National Health and Nutrition Examination Survey (NHANES) recently incorporated questions about weekday and weekend sleep schedules. This survey presents an excellent opportunity for researchers to explore the association between sleep timing and a wide range of health outcomes, which we anticipate may provide valuable insights into optimizing sleep patterns for better health outcomes. Sleep timing pertains to when one initiates sleep, typically referenced to societal norms and personal obligations. Other important metrics, such as sleep regularity, refer to the consistency of one’s sleep and wake times across days, including both weekdays and weekends. The NHANES data, which also include actigraphy data, can be leveraged to examine variations in sleep onset and duration across weekdays and weekends, yielding measures of an individual’s sleep regularity or rhythm.

In contrast, conditions like delayed sleep–wake phase disorder (DSWPD), which feature a shift in the timing of sleep onset and offset (mainly assessed by using data on weekend sleep), provide crucial insight into sleep timing. DSWPD is characterized by sleep and wake times that are substantially delayed relative to societal norms, leading to distress or impairment in social, occupational, or other important areas of functioning. DSWPD, often observed among adolescents and young adults, is characterized by a preference for sleep and wake times that are misaligned with societal demands. People with DSWPD often experience comorbid depression, among other conditions. Through continued research into sleep timing, we can advance our knowledge and improve sleep-related health outcomes (7,24,25). Aligning sleep schedules with societal obligations requires management strategies tailored to each person’s needs and circumstances; however, policy changes such as later school start times could be an effective public health intervention (7).

It is vital to recognize sleep as a fundamental pillar of public health. By prioritizing sleep, addressing sleep disturbances and disorders, and promoting comprehensive approaches that encompass mental health and chronic disease prevention, public health initiatives can enhance the well-being of individuals and communities, as well as ameliorate health disparities among racial and ethnic minority groups. And by understanding the complex interplay between sleep, mental health, and chronic diseases, public health efforts can promote sleep health and improve overall well-being. Multisector efforts among individuals and groups are important for advancing research, implementing effective interventions, and addressing health disparities. Sleep health has multifactorial influences, bidirectional relationships with health outcomes, and individual variations, necessitating comprehensive, concrete solutions. For example, policy makers can promote healthier sleep patterns among teenagers by delaying school start times, as suggested by the American Academy of Pediatrics (7). Furthermore, the implementation of guidelines such as those provided by the National Sleep Foundation and the AASM can serve as practical recommendations for individuals of different age groups to ensure adequate sleep (3–6,8). Such actionable steps taken by policy makers, educators, and health organizations can contribute to enhancing sleep health.

Collaborative efforts among researchers, health care professionals, policy makers, educators, and individuals are essential for developing effective interventions and adopting beneficial policies. By doing so, we can anticipate a deeper understanding of the complex interplay among sleep, mental health, and cognitive functioning across the life span, contributing to improved public health outcomes.
